# Correction to: Tissue-specific and repeat length-dependent somatic instability of the X-linked dystonia parkinsonism-associated CCCTCT repeat

**DOI:** 10.1186/s40478-022-01367-y

**Published:** 2022-04-25

**Authors:** Lindsey N. Campion, Alan Mejia Maza, Rachita Yadav, Ellen B. Penney, Micaela G. Murcar, Kevin Correia, Tammy Gillis, Cara Fernandez-Cerado, M. Salvie Velasco-Andrada, G. Paul Legarda, Niecy G. Ganza-Bautista, J. Benedict B. Lagarde, Patrick J. Acuña, Trisha Multhaupt-Buell, Gabrielle Aldykiewicz, Melanie L. Supnet, Jan K. De Guzman, Criscely Go, Nutan Sharma, Edwin L. Munoz, Mark C. Ang, Cid Czarina E. Diesta, D. Cristopher Bragg, Laurie J. Ozelius, Vanessa C. Wheeler

**Affiliations:** 1 Department of Neurology, Massachusetts General Hospital and Harvard Medical School, Boston, MA USA; 2 Department of Neurology, The Collaborative Center for X-Linked Dystonia-Parkinsonism, Massachusetts General Hospital, Charlestown, MA USA; 3 Center for Genomic Medicine, Massachusetts General Hospital, Boston, MA USA; 4 Program in Medical and Population Genetics, Broad Institute of MIT and Harvard, Cambridge, MA USA; 5 Sunshine Care Foundation, Roxas City, Capiz Philippines; 6 Department of Neurology, Jose R. Reyes Memorial Medical Center, Metro Manila, Philippines; 7 Department of Pathology, College of Medicine, University of the Philippines, Manila, Philippines; 8 Department of Neurosciences, Makati Medical Center, Makati, Philippines

## Correction to: Acta Neuropathologica Communications (2022) 10:49 https://doi.org/10.1186/s40478-022-01349-0

Following publication of the original article [1], the authors identified an error in the author name of Alan Mejia Maza.

The incorrect author name is: Alan Meijia Maza

The correct author name is: Alan Mejia Maza

The author group has been updated above and the original article [1] has been corrected.
