# Supplementary material for: Tissue-specific and repeat length-dependent somatic instability of the X-linked dystonia parkinsonism-associated CCCTCT repeat
Source: Acta Neuropathol Commun. 2022 Apr 8;10:49. doi: 10.1186/s40478-022-01349-0 (PMC8994295; doi:10.1186/s40478-022-01349-0)
Supplement: Supplementary file 1 — Additional file 1. Tables S1–S7. [file 40478_2022_1349_MOESM1_ESM.pdf]

Tissue-specific and repeat length-dependent somatic instability of the X-linked dystonia parkinsonism-associated CCCTCT repeat

Lindsey N. Campion[1,2#], Alan Mejia Maza[1,3#,] Rachita Yadav[1,2,3,4], Ellen B. Penney[1,2], Micaela G. Murcar[1,2] Kevin Correia[3], Tammy Gillis[3], Cara Fernandez-Cerado[5], M. Salvie Velasco-Andrada[5], G. Paul Legarda[5], Niecy G. Ganza-Bautista[5], J. Benedict B. Lagarde[5], Patrick J. Acuña[1,2,5], Trisha Multhaupt-Buell[1,2], Gabrielle Aldykiewicz[1,2], Melanie L. Supnet[1,2], Jan K. De Guzman[5,6], Criscely Go[6], Nutan Sharma[1,2], Edwin L. Munoz[7], Mark C. Ang[7], Cid Czarina E. Diesta[8], D. Cristopher Bragg[1,2], Laurie J. Ozelius[1,2]*, Vanessa C. Wheeler[1,3]*

[#]Equal contribution

*Corresponding authors

[1]Department of Neurology, Massachusetts General Hospital and Harvard Medical School, Boston, MA, USA

[2]Department of Neurology, The Collaborative Center for X-linked Dystonia-Parkinsonism, Massachusetts General Hospital, Charlestown, MA, USA

[3]Center for Genomic Medicine, Massachusetts General Hospital, Boston, MA, USA

[4]Program in Medical and Population Genetics, Broad Institute of MIT and Harvard, Cambridge, MA

[5]Sunshine Care Foundation, Roxas City, Capiz, Philippines

[6]Department of Neurology, Jose R. Reyes Memorial Medical Center, Metro Manila, Philippines

[7]Department of Pathology, College of Medicine, University of the Philippines, Manila, Philippines

[8]Department of Neurosciences, Makati Medical Center, Makati, Philippines

Table S1: Primers and PCR Conditions

Repeat	Primer	Reagent	PCR conditions
XDP SVA Hexamer (Standard sizing)	5’-[FAM]AGCAGTACAGTCCAGCTTTGGC-3′	PrimeSTAR GXL	94°C x 2 min; 30 x (98 °C x 10 s, 64°C x 35 s)
	5′-CTCAAGCCTTATTACAATGCCAGT-3′		
LIPG SVA Hexamer (Standard sizing)	5’-CAGCAGTACAGTCCAGCTTC -3	PrimeSTAR GXL	94°C x 2 min;30 x (98°C x 10 s, 60°C x 15 s, 69°C x 25 s)
	5’-[FAM]AATAGAGTGTATCCGGTGAGC-3’		
XDP SVA Hexamer (Small pool-PCR)	5’-[FAM]AGCAGTACAGTCCAGCTTTGGC-3′	PrimeSTAR GXL	94°C x 2 min; 35 x (98°C x10 s, 64°C x 35 s)
	5′-CTCAAGCCTTATTACAATGCCAGT-3′		

Table S2: **List of XDP patients with blood and postmortem brain tissue available**

Patient	Blood Repeat Length	Brain Repeat Length
17-012	**42**	**43**
17-019	42	42
18-002	41	41
18-003	42	42
18-004	41	41
19-009	39	39
19-012	52	52
19-013	41	41
19-015	36	36
19-017	**44**	**45**
19-018	45	45
19-019	38	38
19-020	43	43
19-021	**37**	**36**
20-024	46	46
20-025	38	38
20-026	39	39
21-027	43	43
21-028	37	37
21-029	42	42
21-031	**47**	**48**

Table S3: XDP individuals with postmortem brain regions used for analyses of XDP CCCTCT repeat instability

Brain ID	Repeat Length	BA9	Cd	Cereb	Cin. Gyrus	DCN	Hip	Insula	ION	Lat. Th	Med. Th	Oc. Cx	Parietal Cx	Pu	RN	SbN	STh	TmP	*N*
16-12	41	X	X	X	X	X	X	X		X	X	X	X	X	X	X		X	15
16-13	46	X	X	X	X	X	X	X	X	X	X	X	X	X	X	X	X		16
17-01	44	X	X	X	X	X		X		X	X	X	X	X	X	X		X	14
17-05	42	X	X	X	X	X	X	X	X	X	X	X	X	X	X	X	X	X	17
17-06	41	X	X	X	X	X	X	X	X	X	X	X	X	X	X	X		X	16
17-10	34	X		X	X	X	X	X		X	X	X	X	X				X	12
17-12	43	X	X	X	X	X	X	X	X	X	X	X	X	X	X	X		X	17
17-13	46	X	X	X	X	X	X	X	X	X	X	X	X	X	X	X		X	16
17-14	36	X		X	X	X	X	X	X	X	X	X	X	X	X	X	X	X	16
17-17	55	X	X	X	X	X	X	X	X	X	X	X	X	X	X	X	X	X	17
17-19	42	X	X	X	X	X	X	X	X	X	X	X	X	X	X	X	X	X	18
18-001	40	X	X	X	X	X	X	X		X	X	X	X	X		X		X	14
18-002	41	X		X	X	X	X			X	X	X	X			X		X	11
18-003	42	X	X	X	X	X	X	X		X	X	X	X	X		X		X	14
18-004	41	X	X	X	X	X	X	X		X	X	X	X	X		X		X	14
18-006	35	X	X	X	X	X	X	X		X	X	X	X	X	X	X	X	X	16
19-007	42	X		X		X						X						X	5
19-008	41	X	X	X	X	X	X	X		X	X	X	X	X		X	X	X	15
19-009	39	X	X	X	X	X	X	X		X	X	X	X	X		X	X	X	16
19-010	39			X															1
19-011	43											X							1

Brain ID	Repeat Length	BA9	Cd	Cereb	Cin. Gyrus	DCN	Hip	Insula	ION	Lat. Th	Med. Th	Oc. Cx	Parietal Cx	Pu	RN	SbN	STh	TmP	*N*
19-012	52			X															1
19-013	41			X															1
19-014	50			X															1
19-015	36			X															1
19-016	43			X															1
19-017	45	X	X	X	X	X	X	X	X	X	X	X	X	X		X		X	16
19-018	45	X	X	X	X	X	X	X		X	X	X	X	X		X		X	15
19-019	38			X															1
19-020	43			X															1
19-021	36			X								X							2
19-022	39			X															1
20-024	46			X															1
20-025	38			X															1
20-026	39			X															1
21-027	43			X															1
21-028	37			X															1
21-029	42			X															1
21-030	46			X															1
21-031	48			X								X							2
21-032	34			X															1
**Total**		21	17	40	20	21	19	19	9	20	20	24	20	19	11	19	8	20	

Brains tissues analyzed are: BA9 = frontal cortex Brodmann area 9, Cd=caudate, Cereb=cerebellum, Cin.Gyrus = cingulate gyrus, DCN = deep cerebellar nuclei, Hip = hippocampus, Insula = insular cortex, ION = inferior olivary nucleus, Lat.Th = lateral thalamus, Med.Th = medial thalamus, Oc. Cx = occipital cortex, Parietal Cx = parietal cortex, Pu = putamen, RN = red nucleus, SbN=substantia nigra, STh = subthalamic nucleus and TmP = temporal pole.

Repeat length (modal allele in standard GeneMapper-based genotyping) in cerebellum except for 19-11 (occipital cortex). Repeat length was identical across brain regions for most individuals; exceptions are shown in Fig. S2.

Table S4: Pair-wise statistical comparisons of XDP CCCTCT expansion indices in blood and brain regions

	P-value																	
	**Blood**	**Cereb**	**Cd**	**SbN**	**ION**	**RN**	**Med.Th**	**Hip**	**Pu**	**Lat.Th**	**DCN**	**STh**	**Cin.Gyrus**	**BA9**	**Parietal Cx**	**Insula**	**TmP**	**Oc.Cx**
**Blood**	-	2e-16	2.2e-09	1.6e-10	7.5e-05	4.5e-06	4.9e-11	1.6e-10	1.6e-10	4.5e-11	1.3e-11	2.8e-04	4.8e-11	1.3e-11	4.5e-11	1.5e-10	4.5e-11	4e-13
**Cereb**		-	ns	ns	ns	ns	ns	0.05003	ns	1.3e-03	4e-03	0.03	1.7e-03	2.7e-05	1.5e-08	1.7e-08	2.6e-09	7.7e-10
**Cd**			-	ns	ns	ns	ns	ns	ns	ns	ns	ns	ns	ns	0.01	0.015	6e-03	1.9e-04
**SbN**				-	ns	ns	ns	ns	ns	ns	ns	ns	ns	ns	ns	0.02	8.5e-03	3. 6e-04
**ION**					-	ns	ns	ns	ns	ns	ns	ns	ns	ns	ns	ns	ns	ns
**RN**						-	ns	ns	ns	ns	ns	ns	ns	ns	ns	ns	ns	0.01
**Med.Th**							-	ns	ns	ns	ns	ns	ns	ns	ns	ns	ns	7e-03
**Hip**								-	ns	ns	ns	ns	ns	ns	ns	ns	ns	0.01
**Pu**									-	ns	ns	ns	ns	ns	ns	0.04	8.5e-03	5.9e-04
**Lat.Th**										-	ns	ns	ns	ns	ns	ns	ns	0.01
**DCN**											-	ns	ns	ns	ns	ns	0.04	1.7e-03
**STh**												-	ns	ns	ns	ns	ns	ns
**Cin.Gyrus**													-	ns	ns	ns	ns	ns
**BA9**														-	ns	ns	ns	ns
**Parietal Cx**															-	ns	ns	ns
**Insula**																-	ns	ns
**TmP**																	-	ns
**Oc.Cx**																		-

Table shows the results of pairwise Wilcoxon rank sum tests comparing expansion indices in each tissue with Bonferroni-corrected P values. Note that the comparisons are not equally powered as the number of samples for each tissue differs (Table S3).

ns = not significant.

Table S5: Frequencies of repeat length changes relative to the modal allele from single molecule SP-PCR data

Repeat length change	Frequency of events (%)								
	17-17				19-008				18-006
	Cd	Pu	Cereb	Oc. Cx	Cd	Pu	Cereb	Oc. Cx	Oc. Cx
≥-35	0.41	0	0.82	0	0	0	0	0	0
≥-30	1.64	0	0.82	0.47	0	0	0	0	0
≥-25	2.46	0	0.82	1.89	0	0	0	0	0
≥-20	2.46	0	1.65	2.84	0	0	0	0.59	0
≥-15	2.88	0	2.47	3.79	0	0	0.7	2.38	0
≥-10	2.88	0	7.43	5.68	0.7	0	0.7	2.97	0
≥-5	4.11	0	9.92	10.9	0.7	0.6	0.7	2.97	0.54
≥-1 (all contractions)	29.21	22.4	45.45	32.23	16.9	23.7	16.31	26.19	17.58
0 (modal allele)	28.39	34.69	24.79	15.63	28.87	26.83	28.37	20.24	24.17
≥+1 (all expansions)	42.38	42.85	29.75	52.13	54.22	49.39	55.31	53.57	58.24
≥+5	2.46	3.4	14.04	18.48	7.74	6.7	4.96	9.52	14.28
≥+10	1.64	1.36	14.04	8.05	0.7	2.43	0	2.97	5.49
≥+15	1.23	1.36	13.22	5.68	0	2.43	0	0	2.19
≥+20	0.41	0	12.39	3.31	0	2.43	0	0	1.09
≥+25	0.41	0	12.39	2.36	0	2.43	0	0	0.54
≥+30	0.41	0	12.39	2.36	0	2.43	0	0	0
≥+35	0	0	12.39	0.94	0	2.43	0	0	0
≥+40	0	0	12.39	0.94	0	2.43	0	0	0
≥+45	0	0	0.82	0.47	0	2.43	0	0	0

Frequencies of repeat length changes relative to the modal allele meeting various thresholds. Negative repeat length changes (contractions) of at least one repeat (≥-1) represent all contraction events, with progressively lower frequencies of contractions of ≥5, ≥10, ≥15, ≥20, ≥25, ≥30 and ≥35 repeats. Positive repeat length changes (expansions) of at least one repeat (≥+1) represent all expansion events, with progressively lower frequencies of expansions of ≥5, ≥10, ≥15, ≥20, ≥25, ≥30, ≥35, ≥40 and ≥45 repeats.

Table S6: XDP individuals with postmortem brain regions used for analyses of *LIPG* CCCTCT repeat instability

Brain ID	Cereb	Cd	Hip	BA9	TmP	Oc. Cx	*LIPG* repeat lengths analyzed [a]
**16-12**	X	X	X	X	X	X	48,56
**16-13**	X						44
**17-01**	X						49,57
**17-05**	X						55
**17-06**	X						61
**17-10**				X			47
**17-13**	X	X	X	X	X	X	45,57
**17-19**	X	X	X	X	X	X	49,60
**18-001**	X						39
**18-002**	X		X	X	X	X	40,67
**18-003**	X						58
**18-004**	X	X	X	X	X	X	53,64
**19-008**	X						50
**19-009**	X	X	X	X	X		49,57
**19-010**	X						56
**19-012**	X						53,60
**19-013**	X						52
**19-014**	X						69
**19-017**	X						53
**19-018**	X						60

**19-019**	X						63
**19-020**	X						52
**19-021**	X						51,61
**19-022**	X						50,55
N	23	5	6	7	6	5	

[a] only one allele is indicated either because a single major allele was detected, indicating homozygosity or because the proximity of the two alleles precluded accurate analysis of expansion peaks of the shorter allele. The repeat length size was determined in cerebellum except for 17-10 brain (BA9).

Table S7: Pair-wise statistical comparisons of *LIPG* CCCTCT expansion indices in brain regions

Brain region	BA9	Cd	Cereb	TmP	Hip	Oc. Cx
**BA9**	-	ns	2.8e-08	ns	ns	ns
**Cd**		-	1.2e-08	ns	ns	ns
**Cereb**			-	6.5e-09	9.7e-09	1.3e-07
**TmP**				-	ns	ns
**Hip**					-	ns
**Oc. Cx**						-

Table shows the results of pairwise Wilcoxon rank sum tests comparing expansion indices in each tissue with Bonferroni-corrected P values.

ns = not significant.
