# Supplementary material for: Tissue-specific and repeat length-dependent somatic instability of the X-linked dystonia parkinsonism-associated CCCTCT repeat
Source: Acta Neuropathol Commun. 2022 Apr 8;10:49. doi: 10.1186/s40478-022-01349-0 (PMC8994295; doi:10.1186/s40478-022-01349-0)
Supplement: Supplementary file 2 — Additional file 2. Figures S1–S5. [file 40478_2022_1349_MOESM2_ESM.pdf]

Tissue-specific and repeat length-dependent somatic instability of the X-linked dystonia parkinsonism-associated CCCTCT repeat

Lindsey N. Campion[1,2#], Alan Mejia Maza[1,3#,] Rachita Yadav[1,2,3,4], Ellen B. Penney[1,2], Micaela G. Murcar[1,2] Kevin Correia[3], Tammy Gillis[3], Cara Fernandez-Cerado[5], M. Salvie Velasco-Andrada[5], G. Paul Legarda[5], Niecy G. Ganza-Bautista[5], J. Benedict B. Lagarde[5], Patrick J. Acuña[1,2,5], Trisha Multhaupt-Buell[1,2], Gabrielle Aldykiewicz[1,2], Melanie L. Supnet[1,2], Jan K. De Guzman[5,6], Criscely Go[6], Nutan Sharma[1,2], Edwin L. Munoz[7], Mark C. Ang[7], Cid Czarina E. Diesta[8], D. Cristopher Bragg[1,2], Laurie J. Ozelius[1,2]*, Vanessa C. Wheeler[1,3]*

[#]Equal contribution

*Corresponding authors

[1]Department of Neurology, Massachusetts General Hospital and Harvard Medical School, Boston, MA, USA

[2]Department of Neurology, The Collaborative Center for X-linked Dystonia-Parkinsonism, Massachusetts General Hospital, Charlestown, MA, USA

[3]Center for Genomic Medicine, Massachusetts General Hospital, Boston, MA, USA

[4]Program in Medical and Population Genetics, Broad Institute of MIT and Harvard, Cambridge, MA

[5]Sunshine Care Foundation, Roxas City, Capiz, Philippines

[6]Department of Neurology, Jose R. Reyes Memorial Medical Center, Metro Manila, Philippines

[7]Department of Pathology, College of Medicine, University of the Philippines, Manila, Philippines

[8]Department of Neurosciences, Makati Medical Center, Makati, Philippines

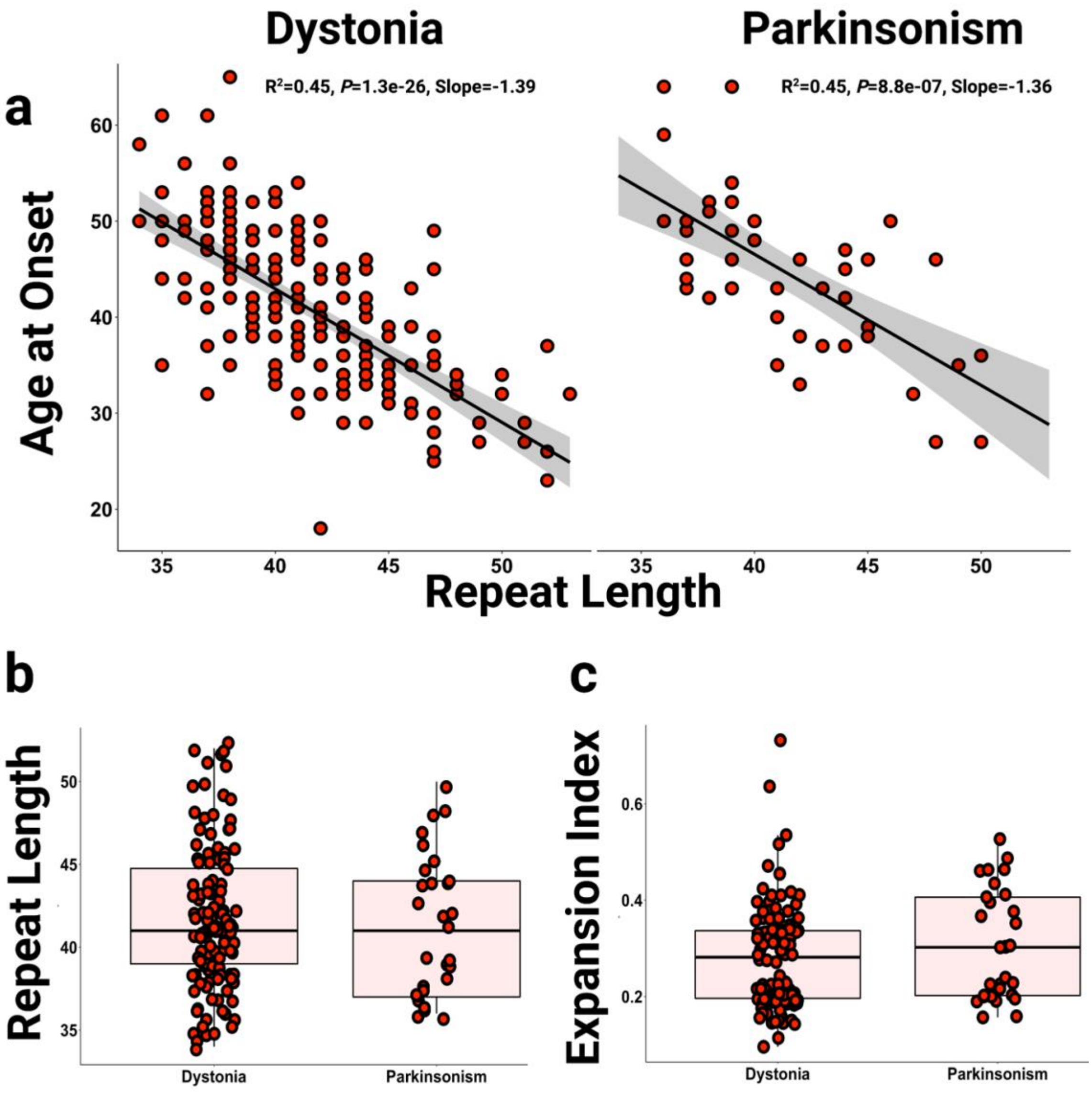

Fig S1. Repeat-related outcomes in individuals exhibiting dystonia or Parkinson's symptoms at onset. a Inverse correlations between CCCTCT repeat length (in blood) with AAO in individuals expressing dystonia (n=194) or parkinsonism (n=43) at onset. Grey shaded areas show 95% confidence interval**. b** Distribution of repeat length (in blood) in individuals expressing dystonia (n=194) or parkinsonism (n=43). **c** Distribution of expansion indices (in blood) in individuals expressing dystonia (n=122) or parkinsonism (n=29). Box-whisker plots show median ± interquartile range (IQR) and red dots show values in individual patient samples. **b-c** showed no statistical differences between groups.

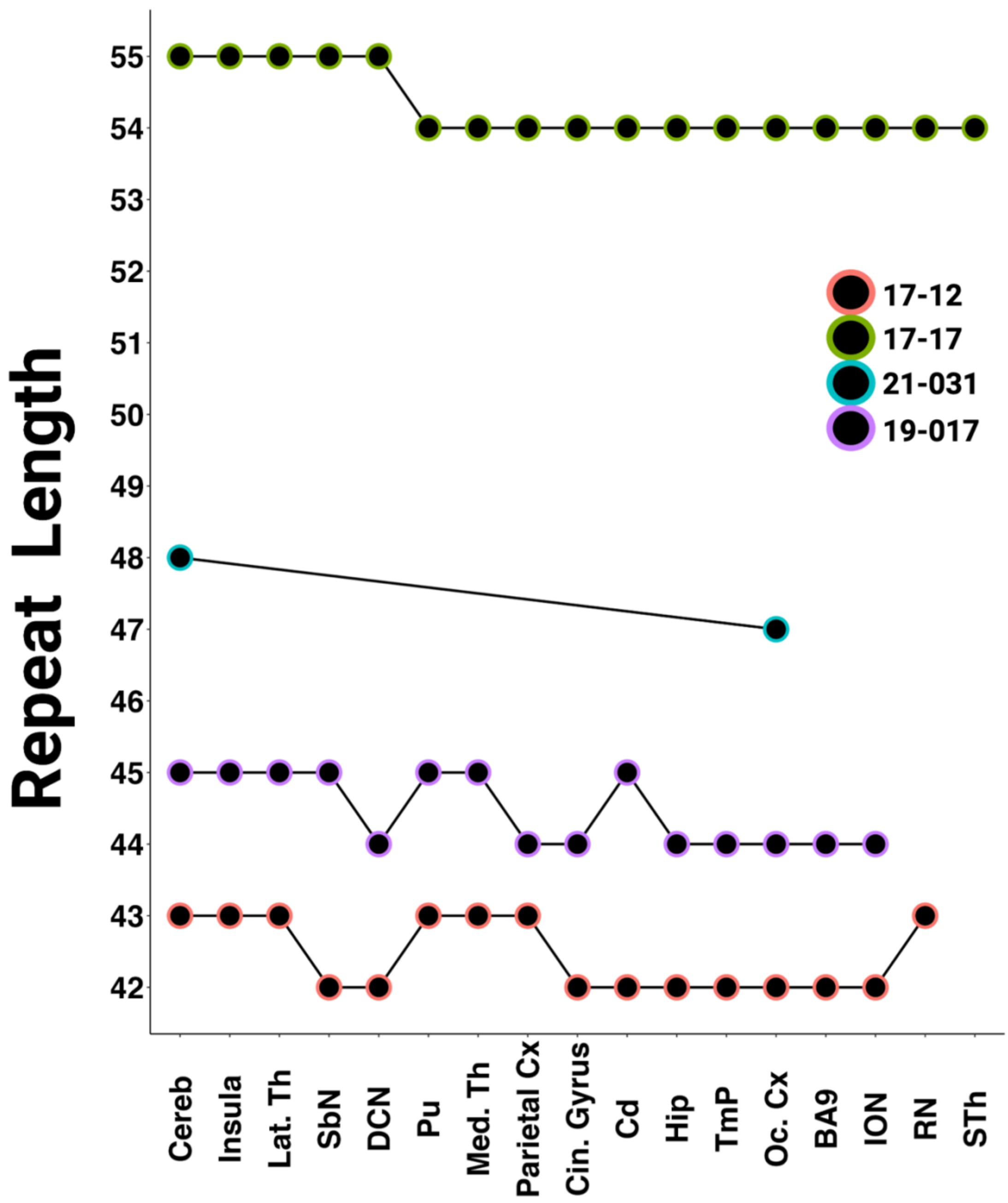

Fig S2: Modal repeat length variation in brain regions. Of the 23 postmortem samples in which multiple brain regions were analyzed, 4 (17-012, 17-17, 19-017 and 21-031) exhibited variation by one repeat unit in the modal repeat length.

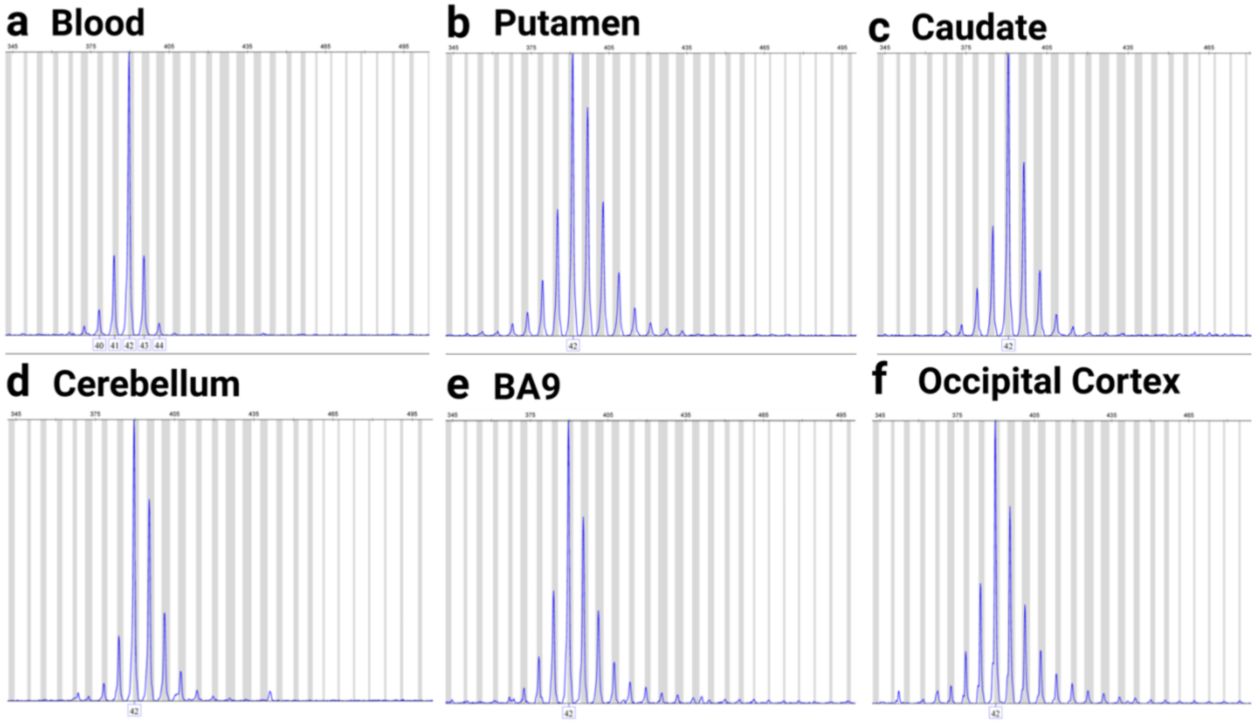

Fig S3: Examples of XDP CCCTCT repeat GeneMapper traces from different tissues. a-f GeneMapper traces of blood and different brain tissues from individual 17-19. “x” axis shows base pair size and peak height respectively. Indicated on the “x”-axes are assigned repeat lengths based on standard repeat controls.

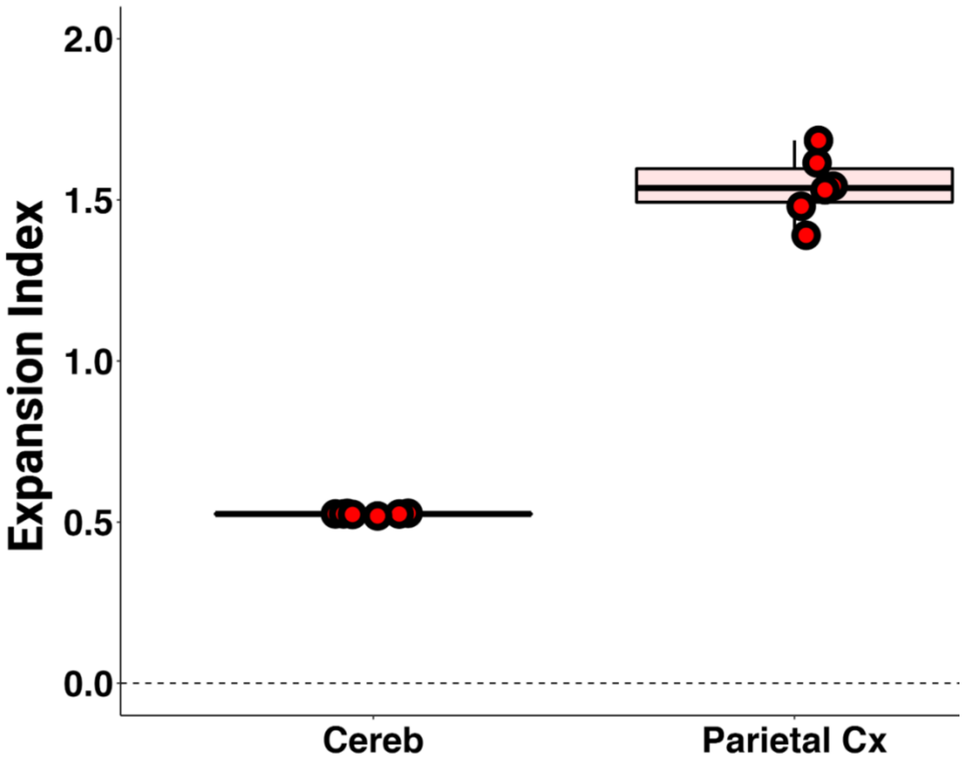

Fig. S4: Technical variability in expansion index values. Expansion indices were derived from 5 independent XDP CCCTCT repeat PCRs from samples 19-022 (Cereb) and 18-001 (Parietal Cx).

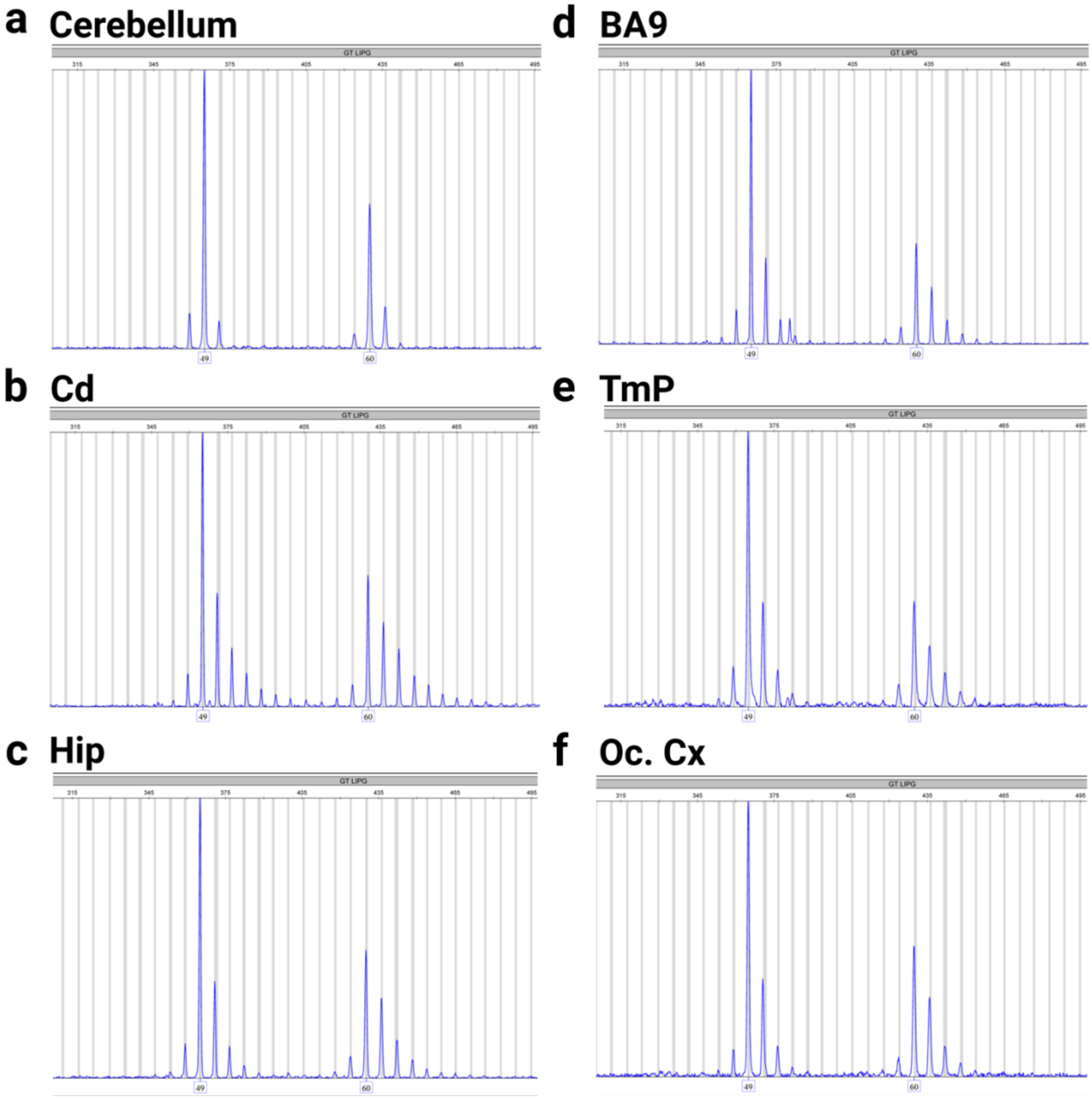

Fig S5: Examples of *LIPG* CCCTCT GeneMapper traces from different tissues.

a-f GeneMapper traces of different brain tissues from individual 17-19. “x” axis shows base pair size and peak height respectively. Indicated on the “x”-axes are assigned repeat lengths based on base pair size.
